# CRISPR/Cas9‐Mediated Correction of the *SLC25A38* Mutation in a Patient With Sideroblastic Anemia

**DOI:** 10.1002/jgm.70114

**Published:** 2026-10-14

**Authors:** Shokoufeh Yazdanparast, Hamid Galehdari, SaeidReza Khatami, Elham Hoveizi, Kaveh Jaseb

**Affiliations:** ^1^ Department of Biology, Faculty of Sciences Shahid Chamran University of Ahvaz Ahvaz Iran; ^2^ Shafa Hospital and Research Center of Thalassemia and Hemoglobinopathy Jundishapur University of Medical Sciences Ahvaz Iran

**Keywords:** CRISPR/Cas9, fibroblast cells, gene editing, mutation, sideroblastic anemia, *SLC25A38* gene

## Abstract

**Background:**

The *SLC25A38* is one of the genes of the heme biosynthesis pathway and disruptive alterations therein cause autosomal recessive congenital sideroblastic anemia (CSA). Primary patient‐derived fibroblasts can be used as a valuable source for disease modeling, cell therapy studies, and the generation of induced pluripotent stem cells. In addition, they are easy to isolate and grow rapidly in vitro. Therefore, effective gene correction in these cells could be an important step towards personalized therapies.

**Methods:**

In this study, fibroblast cells were isolated from a CSA patient and stained, determined by flow cytometry, and detected by immunocytochemistry. Subsequently, transfection optimization was performed with Lipofectamine.

**Results:**

After 24 h of transfection with Cas9/sgRNA3 expression plasmid and single‐stranded oligodeoxynucleotide (ssODN) donor, a transfection efficiency of 49.64% was achieved. The presence of the ssODN donor in the target gene region was examined by PCR‐RFLP and PAGE, observing two fragments with approximate sizes of 116 and 161 bp. These results are consistent with insertion of the ssODN donor into the target gene region in fibroblast cells isolated from the patient.

**Conclusions:**

The findings of this study indicate that the use of the CRISPR/Cas9 system with the designed oligo donor, under the tested conditions, has the potential to produce the expected changes at the target gene locus in patient fibroblast cells. Complementary studies using sequencing, along with examining different transfection conditions, could help more accurately confirm the findings of this study and further optimize this approach for future studies.

## Graphical abstract

In this study, the identity of fibroblast cells isolated from the patient was identified using several methods. Then, the sgRNA3‐pX459 plasmid together with ssODN was transfected into fibroblast cells with a transfection efficiency of 49.64%. Finally, the entry of ssODN donor into fibroblast cells was confirmed using PCR‐RFLP and PAGE methods.

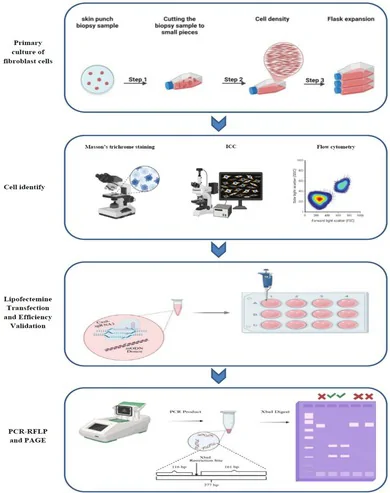

**Abbreviations**
allo‐HCT: allogeneic hematopoietic cell transplant
CSA: congenital sideroblastic anemia
DSBs: double‐strand breaks
GvHD: graft versus host disease
HDR: homology‐directed repair
HLA: human leukocyte antigen
HSPCs: hematopoietic stem and progenitor cells
ICC: immunocytochemistry
iPSCs: induced pluripotent stem cells
PAGE: poly acrylamide gel electrophoresis
PAM: protospacer‐adjacent motif
PCR‐RFLP: PCR–restriction fragment length polymorphism
sgRNA: single guide RNA
ssODN: single‐stranded oligodeoxynucleotide
WES: whole exome sequencing

## 1 Introduction

Sideroblastic anemias are a group of bone marrow disorders, including both genetic and acquired disorders. One of the typical features of this anemia is the presence of ring sideroblasts in bone marrow [1]. Ring sideroblasts are characterized by the pathological iron accumulation within the mitochondria of the erythroid precursor cell and can be seen with Prussian blue staining as a blue ring of granules [2]. Congenital sideroblastic anemia (CSA) can occur either syndromic or nonsyndromic, autosomal or X‐linked, and is associated with heme biosynthesis, Fe‐S cluster assembly or mitochondrial biology gene mutations [3]. CSA due to mutation in the *SLC25A38* gene has been described as one of the causes of severe nonsyndromic autosomal recessive sideroblastic anemia [4]. Patients with mutations in the *SLC25A38* present with microcytic anemia with requirement for regular blood transfusion and iron chelating treatment [5]. Although allogeneic hematopoietic cell transplantation (allo‐HCT) is the only common treatment for this disease, the risk of transplant rejection increases due to graft‐versus‐host disease (GvHD), sensitivity to human leukocyte antigen (HLA) antigens, and reduced survival in the absence of an HLA‐matched sibling donor [6, 7]. Ex vivo gene editing of autologous stem cells and reimplantation of the modified cells overcomes these limitations and may therefore help improve patient outcomes in genetic blood diseases [8]. Advances in engineered nucleases enable precise genome editing to induce DNA breaks through programmed endonucleases including ZFN, TALEN, and CRISPR/Cas9 [9]. Precise genome editing using these nucleases has great potential to advance biological research and develop targeted therapeutics for a wide range of diseases [10]. In this method, after inducing double‐strand breaks (DSBs), the endogenous cellular repair machinery is initiated, and it can be fixed via homology‐directed repair (HDR) [11]. Compared with engineered endonucleases based on DNA‐protein interactions, CRISPR/Cas9, which consists of the Cas9 endonuclease and single guide RNA (sgRNA), has proven to be an effective and simple genome editing tool for targeting genetic modifications [12]. The sgRNA is targeted to genomic regions near a protospacer‐adjacent motif (PAM) and directs the Cas9 endonuclease to cleave the target DNA, causing a DSB 3 bp upstream of the PAM sequence [13]. Genomic regions of mutated cells are excised by the HDR repair mechanism and replaced with a DNA template for the normal gene sequence [14]. There is high potential for disease modeling, gene function dissection and gene editing by CRISPR/Cas9 technology for the treatment of disease. In the clinical section, CRISPR/Cas9 gene editing is promising for monogenic and hematologic diseases and is used mainly in hemoglobinopathy [15, 16]. Given the outstanding potential of CRISPR/Cas9‐mediated genome editing to target pathogenic genetic variants, we aimed to investigate the use of this application to target disease‐causing mutations in patient‐derived cells. Given that a monogenic disorder is caused by a pathogenic variant in the *SLC25A38*, CSA disease could be a good candidate for genetic correction and eventual phenotypic rescue through genome editing. We hypothesized that CRISPR/Cas9‐mediated targeting, in combination with a single‐stranded oligodeoxynucleotide (ssODN) donor template, could drive targeted modification of the mutated genomic locus in patient‐derived cells. To investigate this application, primary fibroblasts derived from an affected patient were used as a cellular model to investigate targeted genome editing. PCR‐based analysis, restriction fragment length polymorphism (RFLP), and polyacrylamide gel electrophoresis (PAGE) techniques were then used to examine the sequence change of interest at the target locus.

## 2 Materials and Methods

### 2.1 Primary Culture of Fibroblast Cells From a Patient With CSA

Skin fibroblasts were obtained from an Iranian patient with CSA, whose symptoms and family history have been previously described [17]. The study was conducted with the approval of the Ethics Committee of Shahid Chamran University of Ahvaz and in accordance with the Declaration of Helsinki protocols. After obtaining written informed consent, a 4‐mm skin biopsy sample was taken. Then, under sterile conditions, the epidermis and dermis of the skin were first separated to expose the desired subcutaneous tissue. After washing the samples, the dermis portion of the skin was cut into smaller pieces. The pieces were placed in culture flasks so that the dermis portion of the samples was exposed to the culture surface. Complete culture medium containing FBS was then added to the tissue pieces and incubated at 37°C and 5% CO_2_.

### 2.2 Determination of Cell Growth Rate

The patient's dermal fibroblast cells at passage 5 were plated in a 24‐well plate. The culture medium was replaced every 2 days. A cell growth curve was obtained by counting the number of cells from day 0 to day 10, every 2 days for each well in triplicate.

### 2.3 Masson's Trichrome Staining

This method was used to detect collagen levels. Fibroblast cells were seeded in a 24‐well plate to reach 70% confluency. Before staining, cells were washed with PBS and fixed with 4% paraformaldehyde for 20 min. Cell staining was performed according to the standard protocol of the Lillehei Heart Institute (Histology and Microscopy Research Facility), University of Minnesota (Minneapolis, MN, USA). The stained samples were observed and examined under a light microscope.

### 2.4 Immunocytochemistry

Fibroblast cells were stained by Immunocytochemistry (ICC). Briefly, cells were fixed with 4% paraformaldehyde. After washing with PBS, cells were permeabilized with Triton X‐100 and then blocked in BSA. Next, the primary antivimentin antibody (Abcam, USA) was added to the cells at a dilution 1:500 and treated overnight at 4°C. After washing with PBS, cells were incubated with Alexa Flour 594 antimouse IgG secondary antibody (1:200, Abcam, USA) for 1 h at room temperature. In addition, cell nuclei were stained with DAPI. Then, the images were detected and analyzed using a fluorescent microscope (Olympus, Japan).

### 2.5 ssODN Donor Design

To facilitate homologous recombination, a 79 nucleotide ssODN donor sequence was designed. In the designed sequence, the mutated T nucleotide was changed to the wild‐type C nucleotide. In addition, the sequence contained two silent point mutations in the PAM region and an XbaI enzyme recognition site provided a convenient way to quickly assess the incorporation of the ssODN sequence at the target locus.

### 2.6 Expression of Fibroblast Surface Markers and Analysis of Transfection Efficiency by Flow Cytometry

Cells dissolved in PBS were incubated with FITC‐conjugated desired surface antibodies. The cells were then examined by flow cytometry (BD FACS Calibur), and the data were analyzed by Flowjo. To examine the transfection efficiency, fibroblasts in third passage were transfected with the pX458 plasmid carrying the sgRNA and oligo donor using Lipofectamine. Then, GFP expression was analyzed by flow cytometry and calculated as the percentage of GFP‐positive cells.

### 2.7 Transfection of Human Fibroblast Cells and Optimization

In order to obtain the highest transfection efficiency, the transfection method was optimized by repeating and making the necessary changes in the amounts of Lipofectamine, plasmid concentration, oligo donor concentration, and transfection time using the pX458 plasmid carrying sgRNA3 and ssODN donor. After optimizing the conditions, appropriate amounts of the pX459 plasmid carrying sgRNA3 and ssODN donor were transfected into fibroblast cells using Lipofectamine according to the manufacturer's instructions. Briefly, 1 day before transfection, fibroblast cells were seeded in a 24‐well plate so that they reached 80%–90% confluency at the time of transfection. On the day of transfection, according to the manufacturer's protocol (Invitrogen), fibroblast cells were transfected. The plate was incubated in an incubator at 37°C and 5% CO_2_.

### 2.8 DNA Extraction, PCR‐RFLP and PAGE

Genomic DNA was extracted from control and transfected fibroblast cells using Promega DNA extraction kit. DNA was amplified by PCR using specific primers around the mutation region. The PCR was performed after an initial denaturation at 94°C for 5 min, followed by at 95°C for 30 s, 58°C for 30 s, 72°C for 40 s (35X), and a final extension step 72°C for 10 min. PCR products of 277 bp surrounding the target site were treated with XbaI enzyme (Thermo Fisher). Then, they were analyzed with 12% denaturing polyacrylamide gels and observed by silver nitrate staining.

## 3 Results

### 3.1 Isolation and Characterization of CSA Patient‐Specific Fibroblast

The steps involved in fibroblast isolation using the explant method are shown in Figure 1. After placing tissue pieces in the culture flasks, the first cell sprouts were visible 7 days later around the tissue. The cells were first round and then changed their shapes and became spindle cells with a vortex arrangement. On the 14th day when the explant pieces were removed from the flasks, many cells with high density were observed around each piece. By changing the medium twice a week and removing nonadherent cells, a relatively homogeneous culture was obtained after 3–4 weeks (one layer of cells with 50%–70% confluency), which is the best time for in situ passage (*P*_o_).

**FIGURE 1:**
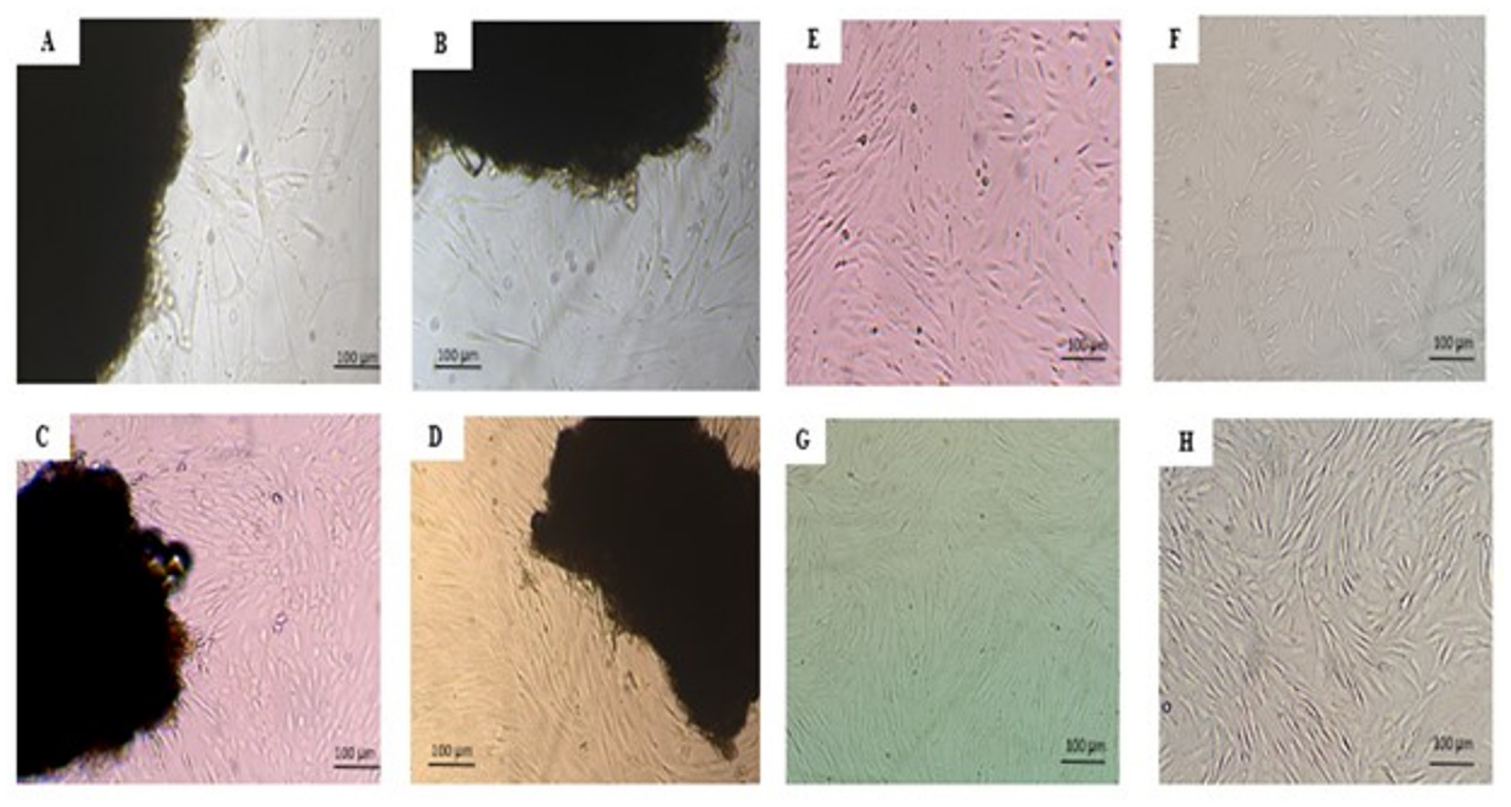
Fibroblast cells derived from a skin punch biopsy of a patient with CSA. (A, B) Tissue pieces and migrated cells from the tissue in the first week. (C, D) Tissue piece and migrated cells from the tissue in the third week. (E, F) Cells after tissue piece removal. (G, H) Cell density and vortex arrangement of cells. Scale bar: 100 μm.

### 3.2 Cell Identity and Viability

Microscopic and staining methods were used to identify cells. In the examination of cells with an inverted microscope, isolated fibroblast cells were observed to be adherent and spindle‐shaped with a vortex arrangement after culture in the third passage (Figure 2A). Evaluation of the proliferation rate of patient dermal fibroblasts at passage 5 shows that these cells exhibit normal growth kinetics from day 0 to day 10 (Figure 2B). In microscopic examination of fibroblast cells isolated from tissue and stained with Masson's trichrome, a significant difference in collagen deposition was observed (Figure 2C). In addition, ICC also confirmed the presence of vimentin in fibroblast cells (Figure 2D). The expression of fibroblast cell surface markers was performed by flow cytometry. The flow cytometry results showed that fibroblast cells significantly expressed markers CD73, CD90, and CD105 (CD73 with 99.9%, CD90 with 98% and CD105 with 99.9%) and the CD34 marker did not have significant expression and was negligible compared to positive markers (Figure 2E).

**FIGURE 2:**
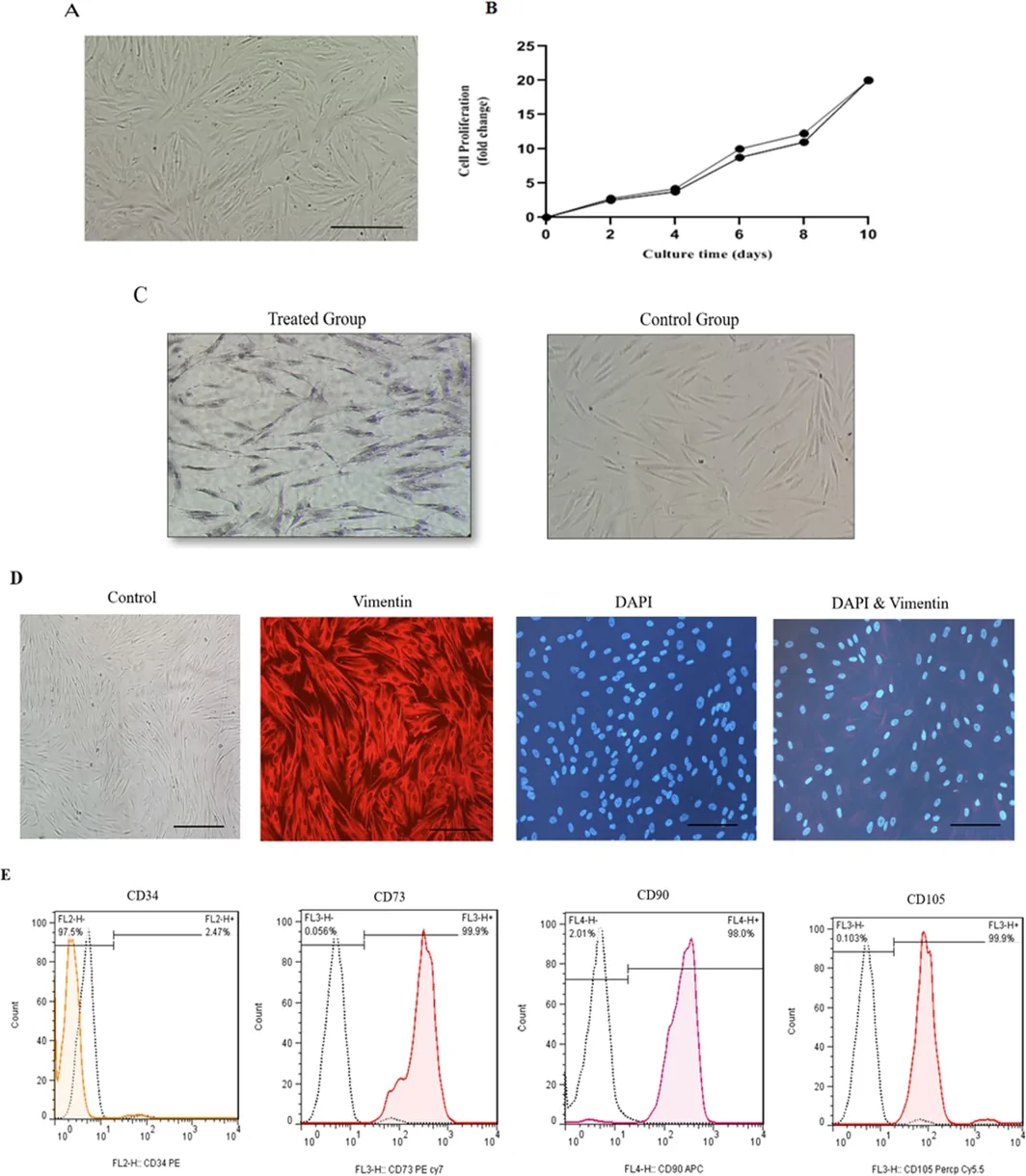
Morphological evaluation and expression profile of surface markers of fibroblasts isolated from patient skin. (A) Third passage fibroblasts show spindle‐shaped morphology under the light microscope (scale bar: 100 μm). (B) Growth curve of fibroblasts isolated from patient skin at passage 5. (C) Examination of collagen in fibroblast cells isolated from the patient using Masson's trichrome staining between the control group (unstained fibroblast cells) and the treated group (fibroblast cells stained with Masson's trichrome staining) using an inverted microscope (scale bar: 100 μm). (D) Immunofluorescent staining of primary fibroblast cells using the vimentin marker. (scale bar: 100 μm). (E) Flow cytometric analysis was used to detect the expression of cell surface markers of third passage fibroblast cells. The cells positively expressed CD73, CD90, and CD105, while negatively expressed CD34.

### 3.3 Oligo Donor Design

To develop suitable donor DNA, we used silent mutations to prevent re‐cleavage of the altered locus by nucleases. The ssODN donor sequence was developed and ordered from Pishgam. The developed ssODN donor sequence is designed as follows:

5′‐CTCCTTTTCCAACCTCT**A**GATCTCCTTAAAACACGCCTG**C**AAACCCT**G**CAGCCCTC**T**GATCATGGgtaggccctgcatt‐3′. The T nucleotide that corrects into the C nucleotide is shown in red and three silent nucleotide changes were made to the ssODN donor sequence, shown in green.

### 3.4 Optimization of Transfection of Fibroblast Cells With Lipofectamine

In order to determine an effective transfection protocol for gene targeting, fibroblast cells were transfected with ssODN donor and recombinant pX458 plasmid carrying sgRNA3 and Cas9 using Lipofectamine according to the manufacturer's instructions. In the first step, four different amounts of Lipofectamine reagent (1, 1.5, 2, and 2.5 μL) were examined. In all steps, the transfection efficiency was evaluated by flow cytometry based on GFP expression. Among the conditions studied, the highest percentage of GFP‐positive cells (22.49%) was obtained in a volume of 2.5 μL of Lipofectamine (Figure 3A). Due to the presence of multiple variables in the transfection process, the amount of Lipofectamine was considered constant and the effect of different plasmid concentrations (130, 220, 450, and 625 ng/μL) was evaluated. The results of flow cytometry analysis showed that the concentration of 220 ng/μL of plasmid, achieving 20.22% of GFP‐positive cells, yielded the highest transfection efficiency (Figure 3B). Then, in order to investigate the optimal concentration of oligo donor, three different concentrations (110, 220, and 440 ng/μL) were selected (the concentrations of other variables were kept constant). The results showed that the addition of oligo donor increased the percentage of GFP‐positive cells compared to the same conditions without donor template, and the highest transfection efficiency (40.95%) was observed at the concentration of 220 ng/μL (Figure 3C). Finally, the effect of the duration of cell exposure to the transfection agent was investigated. For this purpose, after keeping the optimal amounts of Lipofectamine, plasmid, and oligo donor, the level of GFP expression in cells was evaluated at 24, 48, and 72 h after transfection using flow cytometry. Based on the results obtained, the highest transfection efficiency (49.64%) was observed at 24 h after transfection, and GFP expression decreased at 48 and 72 h (Figure 3D). Based on these results, 24 h after transfection was selected as the optimal time point to evaluate the transfection efficiency under the experimental conditions used in this study.

**FIGURE 3:**
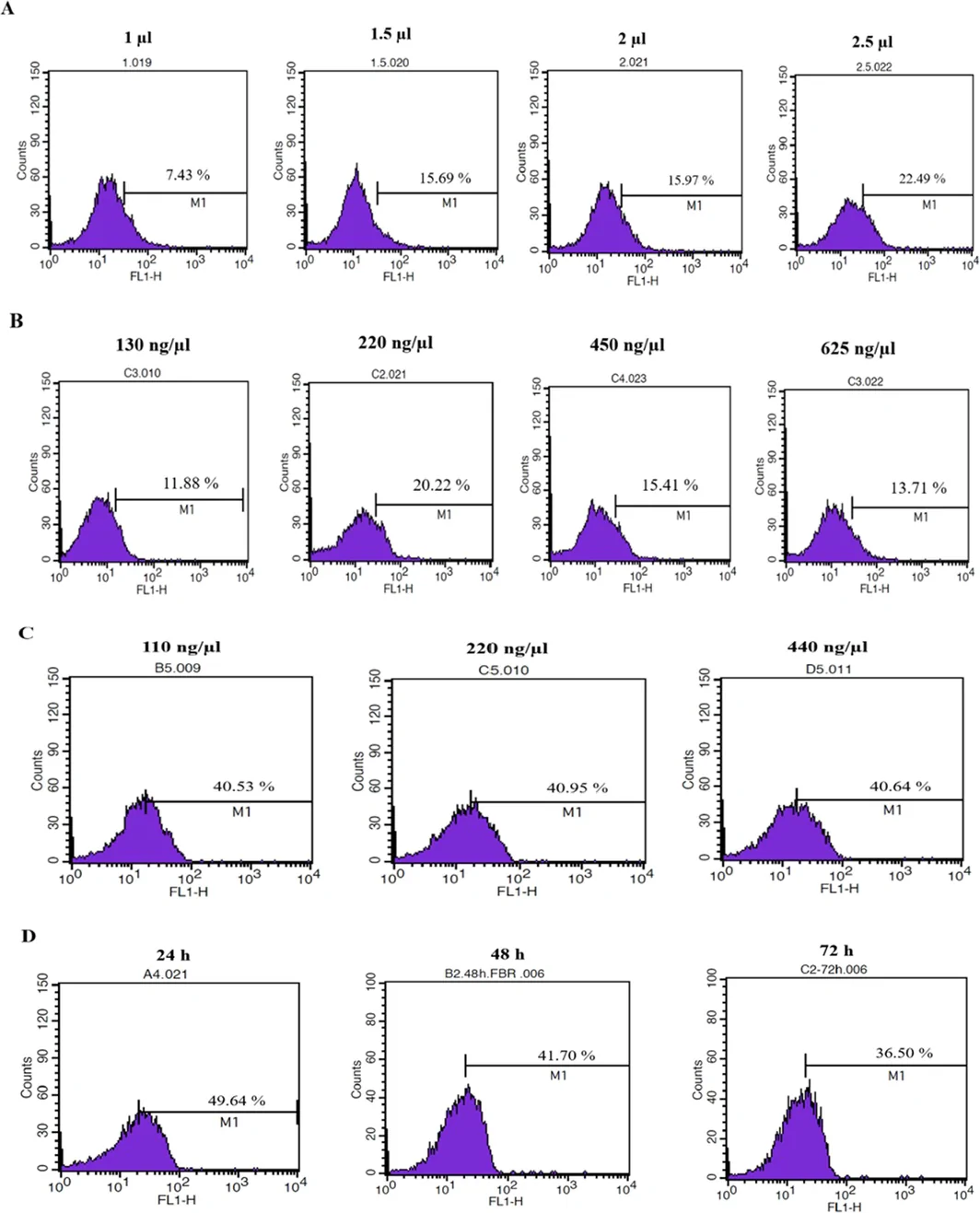
Comparison of flow cytometry results of transfection of fibroblast cells using Lipofectamine. (A) Different amounts of Lipofectamine. (B) Different concentrations of plasmid carrying sgRNA3. (C) Different concentrations of oligo donor. (D) Transfection time.

We then used the same conditions to transfect pX459‐sgRNA3 plasmid in patient fibroblast cells. In order to calculate the lowest concentration of puromycin that would result in complete cell death of fibroblast cells, fibroblast cells were exposed 0.25, 0.5, 0.75, 1, 1.25, 1.5, 1.75, and 2 μg/mL of puromycin before transfection. After 24 h, by examining the cells under a light microscope, the concentration 1.5 μg/mL was determined to be the appropriate concentration for further experiments. The results of the MTT assay also confirmed the above finding (Figure 4).

**FIGURE 4:**
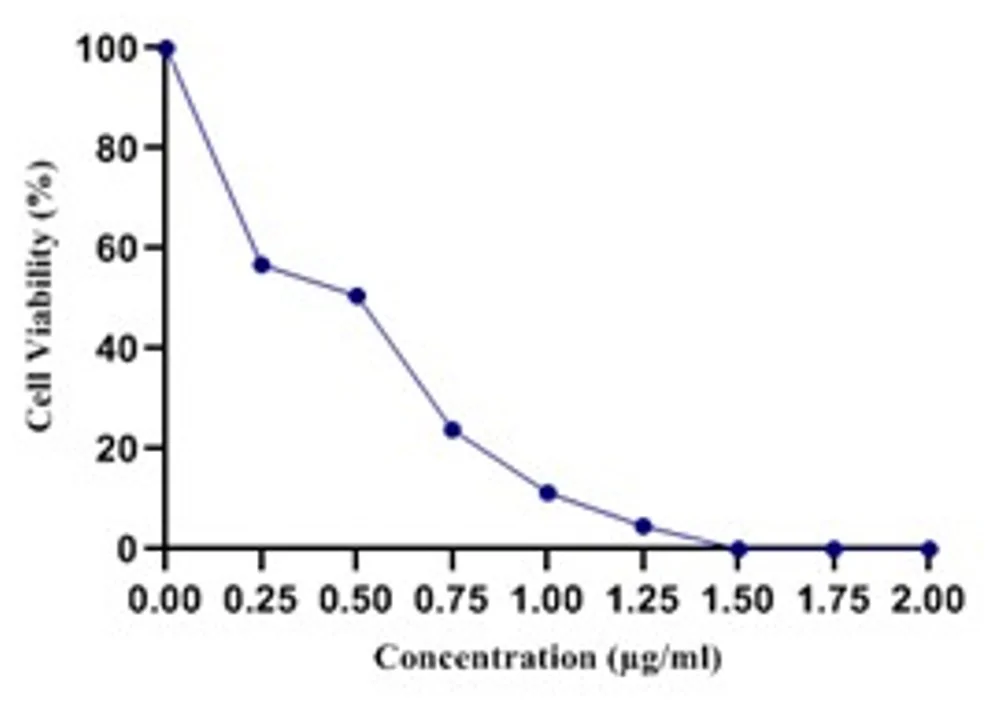
MTT diagram examining the survival of fibroblast cells from the patient in relation to concentrations (0.25, 0.5, 0.75, 1, 1.25, 1.5, 1.75, and 2 μg/mL) of the antibiotic puromycin over a period of 24 h.

### 3.5 Targeting c.166 C>T Mutation in Patient Fibroblast Cells

In order to investigate the presence of the designed oligo donor in the target gene region and to assess the possibility of oligo donor‐dependent modification, after amplification of the target region using PCR, enzymatic digestion of the products with the restriction enzyme XbaI was performed. The products obtained from the enzymatic digestion were examined using PAGE. Among the nine cell populations selected by puromycin, in four samples, a band pattern consistent with the expected change in the target site was observed. As can be seen in Figure 5, in three samples, cleavage was observed in the expected region, which is the cleavage site of the XbaI enzyme, and two fragments with an approximate size of 116 and 161 bp were observed while in the control sample, only the 277 bp was observed.

**FIGURE 5:**
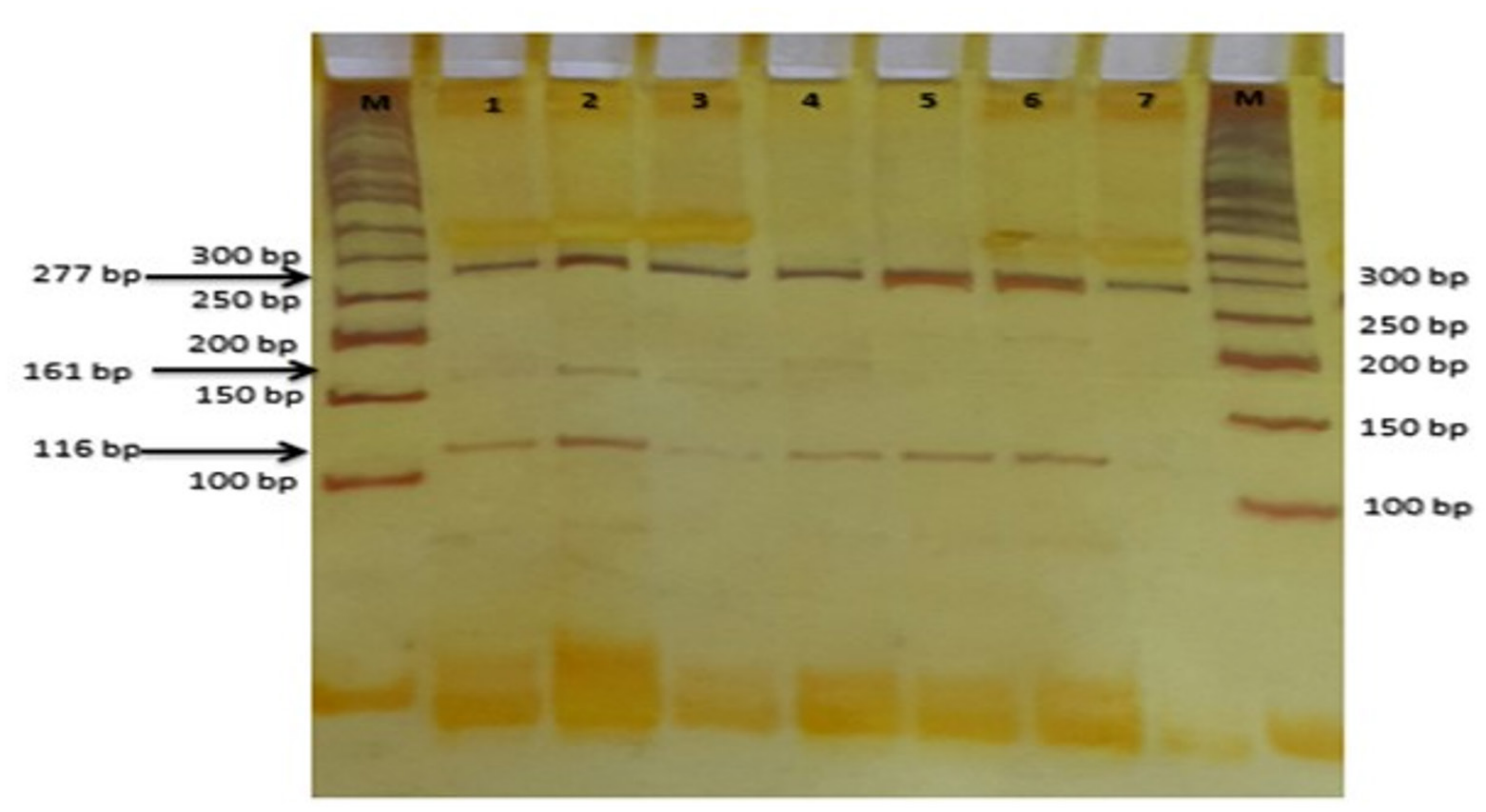
Results of PCR product cleavage with XbaI enzyme to identify the insertion of oligo donor in the *SLC25A38* gene sequence. The presence of two bands at 161 and 116 bp indicates the insertion of oligo donor and the creation of the enzyme cleavage site. The letter M indicates the 50 bp marker, numbers 1 to 6 are the PCR products exposed to the XbaI enzyme, and number 7 is the control sample.

## 4 Discussion

CSA is a rare inherited disorder characterized by the presence of ringed sideroblasts [18]. Significant advances in gene therapy and genome editing, offer an attractive solution for the treatment of blood disorders including CSA [19]. However, due to genetic heterogeneity resulting from defects in various pathways including heme biosynthesis, iron–sulfur cluster biogenesis, and mitochondrial protein function synthesis, its efficacy may vary among CSA subtypes. Some CSA disorders are associated with multisystem manifestations in addition to anemia. In such cases, a gene therapy approach that is limited to the hematopoietic or erythroid compartment may correct the hematologic phenotype but may not fully improve manifestations resulting from other affected tissues. Thus, although erythroid‐directed gene therapy may be a promising strategy for improving anemia in different CSA subtypes, its ability to provide a comprehensive treatment depends on the specific disease‐causing gene, its biological function, and the tissues in which it is expressed. In *SLC25A38*‐associated CSA, which is considered a nonsyndromic form of CSA, the disease is associated with impaired heme biosynthesis in erythroid cells. Given the specific role of erythroid *SLC25A38* in mitochondrial heme biosynthesis, it has been hypothesized that correction of pathogenic *SLC25A38* variants in hematopoietic stem/progenitor cells or erythroid precursors could play a role in restoring erythroid function and improving anemia [18, 20, 21]. Although there have been successful trials in the field of genetically correction of CD34^+^ hematopoietic stem and progenitor cells (CD34^+^ HSPCs), there are still limitations in the clinical use of HSPCs that need to be overcome before clinical use [22, 23]. Therefore, induced pluripotent stem cells (iPSCs), which have pluripotency and unlimited growth in culture, are presented as a suitable option for autologous cell‐based therapy, which can be returned to the patient after genetic correction in order to reconstitute a specific cell line or provide the required protein. This type of cell modeling has been used in proof‐of‐concept and preclinical gene therapy studies for many different diseases [24, 25]. Osborn et al. in 2016 investigated the possibility of generating patient‐derived fibroblast cells for the purpose of individual‐specific cell therapy. They proposed the editing of iPSCs from a FANCI patient at the genomic level to serve as a model for the development of cell therapies [26]. Animal models also shown that the CRISPR/Cas9 system has been utilized successfully to edit the genotype of iPSCs from hemophiliac mice [27, 28, 29]. Skin fibroblasts have characteristics such as easy isolation from skin biopsies, robust growth and proliferation, and cryopreservation, which make them suitable for use in reprogramming [24, 30]. Given that fibroblasts are considered the main cells producing collagen [31, 32], in this study, the presence of collagen in dermal fibroblasts isolated from the patient was examined using Masson's trichrome staining. As observed, the accumulation of collagen in fibroblast cells stained with Masson's trichrome compared to unstained cells showed a significant difference, which can confirm the presence of collagen in fibroblast cells. In addition further studies were conducted on the expression of fibroblast surface markers. CD90 (Thy‐1) is a glycoprotein complex and a fibroblast development marker that binds to the cell surface [31, 33, 34]. Several studies have reported negatively expression for the surface marker CD34 and positively expression for CD73, CD90, and CD105 markers in dermal fibroblasts [35, 36, 37]. The purity of dermal fibroblast cells can also be confirmed by positive staining for vimentin [34, 38, 39]. According to the set of experiments conducted, in this study, the expression level of surface markers CD34, CD73, CD90, and CD105 was measured using flow cytometry and ICC method was used to analyze vimentin in fibroblast cells obtained from patient skin. Characterization of isolated human dermal fibroblasts revealed positive expression of the mesenchymal markers CD73, CD90, and CD105, while CD34, a hematopoietic marker not normally expressed by fibroblasts, was negative. ICC staining also revealed strong expression of vimentin in the isolated cells, which was observed to be red by light microscopy compared with controls. Taken together, the positive expression of CD73, CD90, and CD105, together with the lack of CD34 expression and the vimentin staining, support the identity and relative purity of the isolated human dermal fibroblast population. The results of this study were consistent with previous studies on the importance of continuous expression of these surface markers in skin‐derived fibroblasts.

CRISPR/Cas9 is a novel, simple, and precise system that allows genome editing at the desired location in all cells and organisms [40]. Immune and hematological disorders are among the diseases that could benefit from CRISPR/Cas9 mutation correction, which could be a promising method for making gene changes [41, 42, 43, 44]. Because CSA is a rare disease and few studies have been conducted in these patients, in the present study, after examining the patient sample using whole exome sequencing (WES), a variant in the *SLC25A38* that is associated with the severe form of CSA was identified. Since the disease has an autosomal recessive inheritance pattern, a genetic change in one of the alleles of this gene could be sufficient to maintain protein function. The parents of the patient with the heterozygous genotype had a normal phenotype [17]. Based on the clinical significance of the *SLC25A38*, we designed and evaluated a CRISPR/Cas9 reagent to target the c.166C>T mutation in the of exon 2 of *SLC25A38*.

In our previous study, several sgRNAs designed around the mutation region were evaluated for their ability to induce disruption around the target site. This evaluation was performed using PAGE and sequencing analysis, and the results showed that sgRNA3, which targets a site 13 nucleotides downstream of the mutation site, showed higher efficiency in targeting the target region compared to other sgRNAs tested and was therefore selected as the sgRNA of choice for further experiments [45].

In the present study, with the aim of investigating the possibility of targeted correction of mutation in patient fibroblast cells, the CRISPR/Cas9 system was used alongside a designed ssODN donor as a correction template. In the ssODN donor design, in addition to replacing the wild‐type nucleotide at the mutation‐related position, silent nucleotide changes were also considered. Also, a recognition site for a restriction enzyme was created in the ssODN donor sequence to allow for easier identification and evaluation of possible modified products in subsequent steps. After designing and synthesizing the ssODN donor, patient fibroblast cells were transfected with the ssODN donor and an expression plasmid containing sgRNA3/Cas9 to investigate the possibility of targeted correction of the region containing the mutation. Given that the pX458 plasmid contains a GFP marker, this system was used to investigate and optimize the transfection conditions, and the transfection efficiency was determined based on the percentage of GFP‐positive cells using flow cytometry. The transfection experiment was performed by repeating and varying the experimental variables including the amount of lipofectamine, plasmid concentration, ssODN donor concentration, and time after transfection in order to optimize the reaction conditions. In order to evaluate the effect of each factor, only one of the variables was changed at each stage and the other conditions were kept as constant as possible. The results showed that the highest amount of GFP‐positive cells (49.64%) was obtained in conditions where the ssODN donor and plasmid concentrations were 220 ng/μL and the evaluation of the cells was performed 24 h after transfection. In addition, in the tested conditions, the highest transfection efficiency was observed at concentrations where the plasmid to ssODN donor ratio was approximately 1:1. These results suggested that the 1:1 ratio of plasmid vector to ssODN donor may be associated with higher transfection efficiency under the conditions studied. However, additional experiments using different ratios of vector to ssODN donor are necessary, and examining different ratios in future studies could help determine more appropriate conditions. After optimizing the transfection conditions, the obtained conditions were used to transfect the pX459‐sgRNA3 plasmid into fibroblast cells derived from the patient. Because the pX459 plasmid contains the puromycin resistance gene, this feature was used to select the desired transfected cells. In the next step, RFLP and PAGE methods were used to investigate the expected changes at the target locus. Lichtenstein et al. (2016) utilized the Hinf1 enzyme in detecting homologous recombinant clones after introducing two single‐nucleotide variants within the ssODN donor sequence to create the Hinf1 restriction endonuclease site. Precisely targeted clones were identified by Hinf1 digestion of the patient‐specific PCR product and subsequently confirmed by Sanger sequencing [46]. Abu‐Bonsrah et al. (2016) synthesized ssODN donor with a restriction enzyme site and calculated HDR‐mediated genomic editing frequency by digesting the PCR product with restriction enzymes BamH1 and EcoRV, which gave a range of 34%–66% [47]. In the present study, the XbaI enzyme was used to examine samples with ssODN donor. The PCR products were subjected to digestion with the XbaI enzyme, and in four of the nine selected samples, two fragments with an approximate size of 116 and 161 bp were observed. These results indicated that in a part of the cell population, a change in the target region occurred, which is consistent with the expected pattern related to the ssODN donor design. However, because the samples were pooled cell populations, it is not possible to determine based on these results what proportions of the cells have undergone this change. The results of these studies provided evidence of the expected change in the target region and the observation of a pattern consistent with the desired modification. However, RFLP and PAGE results alone cannot confirm the accuracy of the sequence modification or the full nature of the changes made. Sequencing, especially in positive samples identified by RFLP, will be necessary to accurately confirm the changes made, verify the accuracy of the mutated nucleotide substitution, and determine the status of other possible changes in the target region. Furthermore, in this study, off‐target effects of CRISPR/Cas9 were not experimentally investigated. Although sgRNA design using bioinformatics tools such as CRISPOR is carefully designed to achieve high target specificity and minimize the possibility of off‐target, the possibility of unintended off‐target editing cannot be completely ruled out. Therefore, future studies investigating off‐target effects could contribute to complementing the findings of this study. Finally, this study was conducted using fibroblasts derived from a single patient. Further investigation in cells from other patients will help demonstrate the broader applicability of the present findings. Therefore, these findings were interpreted as preliminary evidence of the establishment of a genetic pattern consistent with the desired modification in selected cell populations, and further studies are needed to conclude on the therapeutic efficacy or clinical applicability of this approach.

## 5 Conclusions

CSA is a severe autosomal recessive nonsyndromic disorder associated with mutation in *SLC25A38*, and most patients require regular blood transfusions. In the present study, we used patient‐derived primary skin fibroblasts as a cellular model to evaluate CRISPR/Cas9‐mediated targeting of the c.166C>T variant in *SLC25A38*. Our findings suggest that the combination of pX459‐sgRNA3 and ssODN donor can target the desired genomic locus in patient‐derived fibroblasts, although DNA sequencing is required to accurately confirm the editing outcome. These results demonstrate that targeting the desired *SLC25A38* region using CRISPR/Cas9 is feasible in this cellular model and provide initial evidence of molecular changes after editing. These findings may provide a basis for future studies aimed at optimizing genome editing strategies and the capacity of CRISPR/Cas9 in CSA.

## Author Contributions

**Shokoufeh Yazdanparast:** conceptualization, investigation, data curation, methodology, formal analysis, writing – original draft, writing – review and editing. **Hamid Galehdari:** conceptualization, project administration, supervision, funding acquisition, writing – review and editing. **SaeidReza Khatami:** conceptualization, project administration, supervision, resource, writing – review and editing. **Elham Hoveizi:** project administration, supervision, resource, writing – review and editing. **Kaveh Jaseb:** resource.

## Funding

The authors disclosed receipt of the following partial financial support for the research, authorship, and publication of this article. Partial financial support was received from Shahid Chamran University of Ahvaz (Grant SCU.SB99.90).

## Ethics Statement

The work described has been carried out in accordance with The Code of Ethics of the World Medical Association (Declaration of Helsinki) for experiments involving humans. All procedures were performed in compliance with relevant laws and institutional guidelines and have been approved by the appropriate institutional committee. Reference number of ethical approval: EE/99.3.02.39634/scu.ac.ir.

## Consent

Written informed consent was obtained from subject family members before inclusion in this study.

## Conflicts of Interest

The authors declare no conflicts of interest.

## Data Availability

The data that support the findings of this study are available from the corresponding author upon reasonable request.
